# Developmental Coordination Disorder and Joint Hypermobility in Childhood: A Narrative Review

**DOI:** 10.3390/children9071011

**Published:** 2022-07-07

**Authors:** Domenico M. Romeo, Ilaria Venezia, Margherita De Biase, Federica Ascione, Maria Rosaria Lala, Valentina Arcangeli, Eugenio Mercuri, Claudia Brogna

**Affiliations:** 1 Pediatric Neurology Unit, Fondazione Policlinico Universitario A. Gemelli IRCCS, 00168 Roma, Italy; eugenio.mercuri@policlinicogemelli.it (E.M.); claudia.brogna@policlinicogemelli.it (C.B.); 2 Pediatric Neurology Unit, Università Cattolica del Sacro Cuore, 00168 Roma, Italy; venezia.ilaria@gmail.com (I.V.); margherita.debiase@gmail.com (M.D.B.); fedeascione@gmail.com (F.A.); mariarosarialala.mrl@gmail.com (M.R.L.); 3 Clinical Psychology Unit, Fondazione Policlinico Universitario A. Gemelli IRCCS, 00168 Roma, Italy; valentina.arca@gmail.com

**Keywords:** developmental coordination disorder, joint hypermobility, outcome

## Abstract

Children with developmental coordination disorder (DCD) and joint hypermobility could present an overlap of symptoms and motor functional difficulties. The link between these two clinical conditions has not yet been clarified. Recent studies reported a high incidence (30–50%) of motor delay in children who are referred to hypermobility and of enhanced joint hypermobility in children with DCD. The aim of this study was to provide a critical review of the literature outlining the association between DCD or limited motor performance and joint hypermobility. Studies were eligible for inclusion if they were written in English and human-based. All the studies were first selected, looking for the presence of a clinical association between developmental coordination disorder or motor performance and hyperlaxity and reporting details of outcome. After a review of the full texts, 16 articles for a total of 1898 children met the inclusion criteria. In general, there was evidence of a higher incidence of motor delay or DCD in children who are referred to hypermobility and of enhanced joint hypermobility in children with DCD with similar range of functional difficulties. These results could influence the way to support children with rehabilitation and the type of intervention according to the prevalence of one of the two conditions.

## 1. Introduction

Developmental coordination disorder (DCD) is a common neurodevelopmental disorder as well as a neuromotor condition, affecting about 5%–6% of school-aged children, more prevalent in males [1], and describing children with motor coordination difficulties. As defined in the American Psychiatric Association’s latest edition of the *Diagnostic and Statistical Manual of Mental Disorders* (DSM-5) [2], children affected by DCD have a motor coordination below expectations for their chronologic age. They are usually described as “clumsy” or “inaccurate” when performing, with possible delays in early motor milestones (e.g., walking); the gross and/or fine motor skill deficits interfere with activities of daily living or in school productivity/academic achievements. Other medical conditions (e.g., neurological condition, visual impairment) or intellectual disability could not explain this motor skill deficit [3,4]. Standardized tests have been developed to diagnose children with DCD and the most used test is considered the Movement Assessment Battery for Children (M-ABC) [5]. The aetiology of DCD remains unknown, despite many hypotheses having been suggested to explain its neurodevelopmental pathogenesis [1,2,3,4]. Most children diagnosed with DCD can present with a variety of functional difficulties (fine and gross motor impairments, reading and socializing impaired proprioception, coordination difficulties, low muscle tone and joint pains). A physical and occupational therapist would be the key professional involved in the rehabilitation programme including a multi-level approach (motor functional skill approach, perceptual-motor therapy, neurodevelopmental and cognitive approach, appropriate orthotics) [6,7].

In the last 15 years, some authors observed functional similarities between children with DCD and those with joint hypermobility, speculating on the multisystem nature of DCD [8,9].

Joint hypermobility or joint laxity or generalized joint hypermobility (all these terms are indifferently used in the literature) is defined as a more-than-normal range of movement (ROM) in a single joint or generalized, i.e., multiple joints [10,11,12,13,14,15,16]; it is usually observed as an isolated phenomenon, defined as asymptomatic hypermobility; in some cases it is also associated with musculoskeletal symptoms such as arthralgia, pain, and extra-articular manifestations (skin hyperextensibility, tendency for osteopenia, gross motor developmental delay); in this case it is described as “joint hypermobility syndrome” or “benign joint hypermobility syndrome” (BJHS), in the absence of other heritable disorders of connective or muscle tissue or other causes of the symptoms.

Children inherently have a greater range of joint motion than adults; the prevalence of hypermobility, as defined by several criteria, varies in different population of children from 5 to 30% [11].

This clinical feature is related to a lower stabilization of joint collagen that occurs as a result of increased cross-linking between adjacent molecules [12].

The Beighton score, based on the analysis of the ROM of all major joints, is one of the most used quantitative measures for analyzing joint hypermobility in children [10]. A physical therapy plays a central role in management of individuals with hypermobility related disorders, with a targeted program aimed improving muscular strength and fitness, correcting motion control of symptomatic joints, use of orthotics and/or sensible footwear, exercises of proprioception, balance manual therapy, tape, hydrotherapy, and relaxation training [7].

Children with joint hypermobility can show a high level (30–50%) of coordination difficulties, with potential widespread functional impact both at home and at school [8].

Therefore, some children presenting with DCD could have joint hypermobility as a cause of their motor and coordination difficulties [8,17,18]; this could influence the type of intervention. It may also potentially highlight the need for greater awareness and education among paediatricians and therapists about joint hypermobility and DCD [8].

The aim of the present narrative review is to increase the amount of knowledge regarding the association between DCD or motor performance and joint hypermobility.

## 2. Methods

### 2.1. Search Criteria

A comprehensive search of the following electronic databases was performed: MEDLINE, EMBASE, PsycINFO, CINAHL. Search terms used were “Joint hypermobility”, “hyperlaxity”, “laxity”, that were each one combined with “developmental coordination disorder”, “dyspraxia”, “motor skills”, “motor performance”.

Duplicates were excluded prior to the retrieval of references. Abstracts for each reference were obtained and screened using the following criteria.

### 2.2. Inclusion Criteria

Studies were eligible for inclusion if they were written in English and human-based. All the studies were first selected, looking for the presence of a clinical association between developmental coordination disorder or motor performance and hyperlaxity and reporting details of outcome. No publication date limits were set. Furthermore, as the onset of symptoms is in the early developmental period and a definite diagnosis of DCD does not usually happen until at least 4 years old, we included studies with an age range of 4–18 years.

### 2.3. Exclusion Criteria

Studies were excluded if they were case reports or if they assessed progressive and/or neurodegenerative disease or well-defined genetic disorders.

### 2.4. Data Extraction and Analysis

The title and abstracts of the studies were independently examined for suitability by two authors (D.M.R., I.V.) and critically checked by a third independent reviewer (C.B.); conflicting viewpoints were discussed until consensus was reached.

For each paper, we considered how the DCD/motor function and joint hypermobility were assessed (clinical evaluation, clinical history or questionnaires). The selected papers were further subdivided into [1] studies assessing joint hypermobility and motor performance/coordination by using M-ABC; [2] studies assessing joint hypermobility and motor performance/coordination by using different kinds of evaluations

## 3. Results

**Figure 1:**
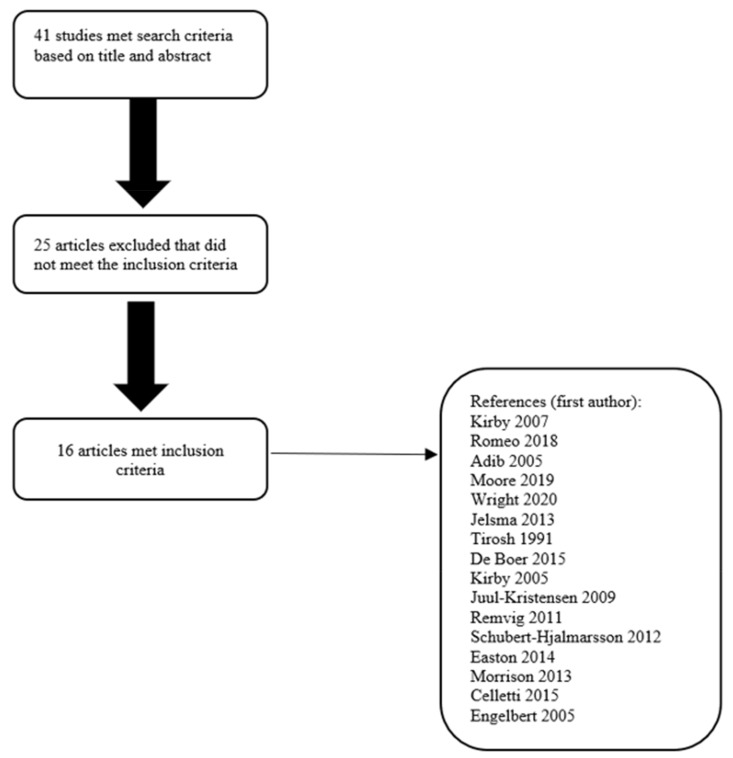
Flow chart for process of article inclusion [8,19,20,21,22,23,24,25,26,27,28,29,30,31,32,33].

A total of 41 studies were initially identified (Figure 1); after a review of the full text, 25 were excluded, as they did not provide details of association between DCD or motor performance and joint hypermobility.

A total of 16 articles [8,19,20,21,22,23,24,25,26,27,28,29,30,31,32], for a total of 1898 children, met the inclusion criteria. Table 1 reports the list of the selected papers with details of the population studied and outcomes.

In nine studies, DCD and motor function were assessed by using the M-ABC, a test used to evaluate motor impairment and to assess motor competence in children [8,19,22,23,25,26,30,32,33]; in five of these nine articles [19,22,23,25,32], joint hypermobility was assessed by using the Beighton scale; in one [33] the authors use the Bulbena criteria; and in three studies [8,26,30] the authors considered a clinical evaluation or questionnaire for hyperlaxity not otherwise specified.

In the remaining seven studies [20,21,24,27,28,29,31], the motor performance was assessed by using different kinds of evaluations such as clinical history, self- or parent-reported questionnaires, clinical examination, or motor competence tests. Among these articles, hypermobility was assessed with the Beighton score in four of seven studies [20,21,27,28].

### 3.1. Studies Assessing Joint Hypermobility and Motor Performance/Coordination by Using the M-ABC

Nine studies reported data on joint hypermobility and motor performance/coordination by using the M-ABC [8,17,22,23,25,26,30,32,33].

In 2018, Romeo et al. studied 132 low-risk preterm infants at pre-school age using the Beighton score and the M-ABC-2 [19]. In this cohort of children, a total of 20% reported joint laxity and achieved significantly lower scores than those without joint laxity on both total scores and subscores of M-ABC-2; no significant age or sex differences were noted.

In 2005, Kirby et al. [26] studied a population of 126 school age children, 68 with BJHS (no test performed) and 58 with DCD (<5th percentile on the M-ABC). The authors found that children with BJHS had a range of functional difficulties that impact on fine and gross motor functions; both children with BJHS and DCD had a similar range of functional daily difficulties. The same authors, in 2007 [8], measured the level of symptoms consistent with JHS into two groups of school-age children, the first one with a diagnosis of DCD (<5th percentile on the M-ABC) and the second group with typically developing children (TDC). The authors reported that the 37% of those with DCD had symptoms of JHS such as pain in joints and pes planus, compared with 7.4% in the TDC group.

Jelsma et al. [23], in a case-control study enrolling 352 healthy controls versus 36 children with DCD, showed that in the DCD group the prevalence of joint hypermobility (evaluated with Beighton scale) was higher (28% vs. 6%) with a significant negative correlation between the M-ABC total score and the degree of hyperextension of the knees.

Celletti et al. [32] studied 41 Italian children with DCD, diagnosed according to DSM-IV and the M-ABC; they further investigated the prevalence of joint hypermobility using the Beighton score. A total of 46% of children with DCD reported joint hypermobility and showed a significant excess of frequent falls and easy bruising than those children with no hypermobility.

Easton et al. [30] studied the relationship between joint hypermobility and motor control in an interventional study of BJHS in childhood. The population was composed of 119 children with documented joint hypermobility, musculoskeletal pain or dysfunction. Motor ability was assessed using the M-ABC-2 and joint hypermobility was assessed by a paediatric rheumatologist (no information about the laxity assessment method was reported). Among the children with BJHS, 32.8% scored ≤15 percentile on the M-ABC 2 (*p* < 0.001).

The last three studies reported no clear relationship between motor performance and joint hypermobility [22,25,33]. De Boer et al. [25] studied a population composed of 249 Dutch children to determine the prevalence of generalized joint hypermobility (using the Beighton scale) and to examine the association with motor performance assessed by the M-ABC-2 and development over time. No significant association between generalized joint hypermobility and total motor performance was reported. In a recent article published in 2020, Wright et al. [22], investigated differences in hypermobility, measured by the Beighton scale, in 60 children across different levels of motor ability assessed using the M-ABC-2. They found no group differences in hypermobility according to the motor competence groups, as a similar Beighton score was reported in children with both normal and abnormal score at the M-ABC-2.

Engelbert et al. reported on 56 children (4–12 years) with joint hypermobility with or without musculoskeletal complaints assessed using the Bulbena criteria; they performed the M-ABC, but no significant association between the presence of a delay in motor development and Bulbena score was found; joint hypermobility was reported in children both with and without severe delay in motor performance [33]

### 3.2. Studies Assessing Joint Hypermobility and Motor Performance by Using Different Kinds of Evaluations

In seven studies [20,21,24,27,28,29,31], the motor performance was assessed by using different kinds of evaluations.

In 1991, Tirosh et al. [24] studied a population of 59 infants aged 18 months by general clinical assessments to screen hypermobility and motor delay; they were further re-assessed at 4.5–5 years of age by using specific tests for gross and fine motor performances. Both gross and fine motor performances were significantly delayed in the group of children with joint hypermobility and motor delay at 18 months. No significant delay was evident in those with joint hypermobility only.

Schubert-Hjalmarsson et al. [29] described hypermobility, balance, pain, activity and participation in children with BJHS and compared these characteristics with those of TDC. Children with BJHS had significantly more hypermobile joints, more pain, scored lower in the balance test and their activity was more affected on a daily basis then TDC.

In 2005, Adib et al. [20] reported on a cohort of 125 children with BJHS (assessed by Beighton scale), recruited from a paediatric rheumatology hypermobility clinic with historical details of developmental milestones, musculoskeletal or soft tissue diagnoses and symptoms. Children reported arthralgia in 74% and were noted to be clumsy in 48% or had poor coordination in 36%; furthermore, 40% had experienced problems with handwriting tasks and 48% had major limitations of school-based physical education activities. The authors concluded that clumsiness, poor coordination and late walking represented difficulties with fine and gross motor development and may have been related to the central nervous system or proprioceptive control.

Morrison et al. [31] reported clinical findings of foot posture and lower limb hypermobility in 14 children with DCD using foot orthoses. A pes planus foot posture and lower limb hypermobility were observed in children with DCD and were at the top end of the scale for lower limb hypermobility. The trend in the data offers preliminary support for podiatric intervention in children with DCD.

The last three studies reported no clear relationship between motor performance and joint hypermobility [21,27,28].

Remvig et al. [28] studied a population of 315 children in a Copenhagen municipality. The study demonstrated a prevalence of generalized joint hypermobility from 11 to 36% according to the Beighton score cut-off, with no gender difference. No correlation between the Beighton score and motor competence tests were reported; increased pain or frequency of injuries were not related to generalized joint hypermobility, and children with level of ≥6 positive Beighton tests performed better in motor competence tests.

In 2009 Juul-Kristensen studied the prevalence of generalized joint hypermobility (BJHS) and motor function assessmentsin 524 Danish children in the second grade [27]. Static balance was better in children with generalized joint hypermobility such as the speed and hand reaction tests. The prevalence of generalized joint hypermobility was from 10 to 29% according to the Beighton cut-off used, with no gender difference. The BJHS group did not perform worse than the non-BJHS group in any of the motor competence tests. There was no significant effect of any of the generalized joint hypermobility groups in the parameters of physical activity level (PAL) and no correlation between number of positive Beighton tests and PAL.

A case-controlled cross-sectional study explored the prevalence of generalized joint hypermobility (Beighton score of ≥6/9) in 73 children, 32 attending physiotherapy and 41 TDC, [21]. In this study, the authors used self-reported or parent-reported questionnaires to evaluate the Physical Activity and the presence of DCD. The prevalence of generalized joint hypermobility was 21.9% of children attending physiotherapy and 17.1% of the school group with no significant gender differences; probably DCD (pDCD) was observed in 72% of the physiotherapy group and 22% of the school group. There was a significantly higher prevalence of pDCD in the physiotherapy group. There was no significant difference in the number of children with pDCD in those with and without generalized joint hypermobility in the physiotherapy group. However, the limited sample size and the use of a subjective measure of motor control reduced the value of the conclusions of the study.

## 4. Discussion

Joint hypermobility is considered a clinical phenomenon that is relatively common during childhood and is usually not related to symptoms requiring medical attention. However different studies have underlined a possible association with chronic health complaints in a small proportion of children, such as pain, musculoskeletal disorders and motor delay; these symptoms could cause a great deal of anxiety in both family and health professionals, requiring the utilization of significant time and resources [8,20]. In 2012, Clarke and Khattab identified few primary research studies related to an association between joint hypermobility and DCD, with non-conclusive evidence due to the limited research papers included in the review (n = 5). More recently, a relationship between joint hypermobility and DCD has been further proposed with variable response rate to physiotherapy, occupational therapy and podiatry according to the prevalence of one of the two conditions [21].

Most of the studies reported in the present review described a clear relationship between motor performance and joint hypermobility [8,19,20,23,24,26,29,30,31,32]. The population was mainly composed by children attending school (primary and secondary school). Among them there were children with DCD and joint hyperlaxity, including patients diagnosed with benign joint hypermobility syndrome.

Different studies used the M-ABC to assess the DCD and the Beighton score to assess hypermobility reporting lower scores on the M-ABC in children with joint laxity [8,19,23,26,30,32].

Other studies used different and not specific clinical tools to assess DCD and hypermobility supporting a non-casual association between DCD and joint hyperlaxity [20,24,29,31]; in these studies, an overlap of symptoms and functional difficulties between the two conditions, such as pain, frequent falls, or flat feet and fine and gross motor function difficulties, were also identified.

The association between joint hypermobility and DCD could be explained by a common alteration in the central nervous system or proprioceptive control [20]. In the developing child with joint hypermobility, it has been proposed a generalized lack of proprioception that may affect the process of organization of spatial and temporal concepts [32]. The further relatively high rate of learning difficulties, dyslexia and dyspraxia suggest the possibility of a central nervous system involvement in these conditions [34]. It has been argued that perceptual difficulties in visuo-spatial processing are the cause of the motor problems in DCD [35,36]. Many children with DCD show poor postural and balance control, especially in extremely difficult situations. The characteristics of this poor control are likely to be task-dependent and the availability of sensory information could influence the quality of postural and balance control, which may be related to proprioceptive errors or some cerebellar dysfunctions [17].

Children with DCD have specific impairments in somatosensory and motor performance tests; joint laxity could influence motor competences due to a reduced proprioception from the joints and suboptimal strength of the muscles that lead to a poor control of joint movement and instability [14,15,16,17,18]. Proprioception is a specialized variation of the sensory modality of touch that includes the sensation of joint movement (kinesthesia) and joint position (joint position sense) [13].

Several studies associated joint hypermobility to an alteration of proprioception [14,15] and to postural stability deficits that might lead to a poor control of joint movements and instability [16]. On the other hand, children with DCD showed an altered postural muscle activity that may contribute to a poor stability [17,18], possibly related to an inconsistent timing of muscle activation sequences, co-contraction and lack of automatization and slowness of response. These characteristics lead to a difficult control of hypermobile joints, since a lack of co-contraction and slowness of response will result in decreased and less well-timed stability of the loaded joints. Having to deal with larger degrees of freedom in joints can co-occur with motor problems in children with DCD [33].

Therefore, according to the results of these studies, two possible subsets of children with joint hypermobility could be identified: one group with normal motor development and no residual motor dysfunction and the other group with joint hypermobility and gross and fine motor dysfunction which may have its origin in the central nervous system. Children with both joint hypermobility and DCD could require a different clinical approach with a prolonged neurodevelopmental follow up compared to children with one single problem; this could be the reason why some children respond differently to the same approach [26]. Podiatric intervention, for example, could be also proposed in these children [31]. Furthermore, children’s families must be involved in this process as home treatment improves the outcome [32].

Among the studies reported in the present review, some of them did not identify a clear correlation between DCD and joint hyperlaxity [21,22,25,27,28,33]. Moreover, few of them reported a negative correlation between the two conditions, showing that balance and motor performance were better in children with joint hypermobility [27,28]. However, the wide age range and the different basic characteristics of children included in these last studies besides using different methods for testing motor competence may have influenced the results [20,33]. Furthermore, it may be possible that children with joint hypermobility and good motor competence in infancy will develop a poor motor performance in adolescence, due to the increasing physical demands over the years [11,28].

In considering the validity of our conclusions, the potential effects of some methodological limitations should be considered that may have affected the analysis of the reviewed papers.

As the present study was not structured as a meta-analysis, each paper was not critically evaluated with no specific and statistical combination of the results of all the studies reviewed.

First, there is a difference in the sample size according to age and geographical origin. Second, the majority of the articles take little account of gender differences without analyzing in depth the differences in symptoms between male and female patients. Third, children included in some studies may have coexistent medical conditions, such as hereditary connective tissue disorders that were not systematically assessed and could impact on motor coordination. As a consequence, DCD may not be directly caused by the joint hypermobility itself, but it may be included in a wider cohort of symptoms.

Another limitation is that different types of evaluations and questionnaires have been used in the studies to evaluate both the motor performance and the joint hypermobility, with a difficult comparison among the studies. Furthermore, most of the studies defined the presence of DCD based on motor assessment tools only; no specific mention on the interference with activities of daily living or in school performance was reported to confirm the diagnosis of DCD.

In conclusion, although the presence of these limitations, the present review point-out the evidence of a higher incidence of motor delay in children who are referred to hypermobility (33%–48%) and of enhanced joint hypermobility in children with DCD (28–46%) with similar range of functional difficulties [20,23,26,30,32]. Highly prevalent, still poorly defined, multisystem disorder in DCD children with joint hypermobility should be considered. The knowledge of these clinical manifestations could be of great interest for carers in term of diagnosis and treatment [6,7]. A poor motor competence and musculoskeletal joint symptoms may interfere with daily life activities, resulting in a more inactive lifestyle in children [28,37].

Children originally assessed for DCD should be investigated with a standardized Beighton protocol using a goniometer for related symptoms (such as laxity or pes planus foot posture); on the other hand, motor performance and coordination (using the M-ABC-2) should be examined in children with hypermobility, as this may influence the type of support given.

Further research regarding the genetic aspects of these disorders and a more in-depth definition of DCD are required in the prognosis description and in the counselling of families.

## Figures and Tables

**Table 1:** Study characteristics.

Ref.	Author, Year	Type of Study	Sample N	Age of Assessment in Months (m) or Years (y)	Joint Hypermobility and Developmental Coordination Disorder	Assessment for DCD/Motor Performance	Assessment for Hypermobility	Others Instruments Used
[8]	Kirby, A. et al., 2007	Case-Control	54	Control group: 5–18 years Case group: 9–17 years	Similar clinical features between JHS and DCD	M-ABC	Questionnaire	Questionnaire covering a range of symptoms consistent with a diagnosis of JHS and related autonomic nervous systemic symptoms
[19]	Romeo et al., 2018	Longitudinal	132	From 12 months to 4 years	Children with joint laxity had lower scores than those without joint laxity on both total scores and subscores of M-ABC-2.	M-ABC-2	Beighton Score (>4)	Touwen Infant Neurological Examination
[20]	Adib, N. et al., 2005	Prospective and Retrospective	125	3–17 years	Children with JHS present Joint pain and coordination problems in 36%	Clinical history	Beighton Score (≥4)	-
[21]	Moore, N. et al., 2019	Cross-sectional, case-control, observational	73	6–12 years	No significant difference in the number of children with pDCD in those with and without GJH.	Self-reported questionnaires Children: Physical Activity Questionnaire for older children Parent: Developmental Coordination Disorder Questionnaire	Beighton Score (≥6)	-
[22]	Wright, K.E. et al., 2020	Cross-sectional observation	60	6–12 years	Hypermobility failed to explain significant variance in motor competence beyond that explained by neuromuscular performance.	M-ABC-2	Beighton score (>7) Lower Limb Assessment Score	Resistance Training Skills Battery for Children (RTSBc), 5-repetition maximum (5RM) leg press and Biodex dynamometry
[23]	Jelsma et al., 2013	Case-control	Case group: 36 Control group: 352	Case group 7–10 years Control group 3–16 years	In the DCD group the prevalence of hypermobility was higher and there was a significant negative correlation between the m-ABC total score and the degree of hyperextension of the knees.	M-ABC M-ABC-2	Beighton score (≥5 for 3–9 years; ≥4 for >10 years)	-
[24]	Tirosh et al., 1991	Longitudinal	59	54–60 months	Both gross and fine motor performance were significantly delayed in the group of children with joint hypermobility and motor delay.	Gross motor performance. Parent perception of motor proficiency:	Clinical evaluation of the mobility of joints	-
[25]	De Boer et al., 2015	Prospective	249	Mean 5.5 years	No significant association was found between GJH and total motor performance.	Bayley Scales of Infant Development, Second Edition M-ABC-2	Beighton Score (≥4; ≥5; ≥6)	
[26]	Kirby et al., 2005	Cohort	126	8–9 years	Children with BJHS present functional difficulties that impact on fine and gross motor function. Children with BJHS and DCD have a similar range of functional difficulties.	M-ABC	Questionnaire	Hypermobility Syndrome Association developmental coordination disorder questionnaire
[27]	Juul-Kristensen, B. et al., 2009	Cross-sectional	349	(8.40 ± 0.52) years	Static balance and speed reaction tests better in children with GJH The BJHS group did not perform worse than the non-BJHS group in any of the motor competence tests. There was no significant effect of any of the GJH groups in the parameters of PAL; no correlation between number of positive Beighton tests and PAL.	Clinical examination and motor competence tests	Questionnaire Beighton score (>4; >5; >6) Brighton Tests	Questionnaire with 75 items on health and physical activity
[28]	Remvig, L. et al., 2011	Cohort	315	10 years	Increased pain or frequency of injures were not related to GJH. Insignificant increased jump height by girls with joint hypermobility. Significant shorter hand reaction time in boys with GJH6.	Motor competence tests:	Beighton score (>4; >5; >6)Brighton Tests	Questionnaire with 75 items on health and physical activity
[29]	Schubert-Hjalmarsson et al., 2012	Cross-sectional	Case group: 20 Control group: 24	8–15 years (11.2 ± 1.9) 8–15 years (11.4 ± 2)	Balance is decreased in children with HMS compared with healthy controls.	Bruininks-Oseretsky test of motor proficiency (balance) Positive association	Del Mar scale	Frequency of Partic- ipation Questionnaire Physical activity was reported in an activity diary
[30]	Easton, V. et al., 2014	Interventional	119	5–16 years	Among the children with BJHS assessed, 32.8% scored ≤15 percentile on the M-ABC (*p* < 0.001).	M-ABC-2	Clinical evaluation	Childhood Health Assess- ment Questionnaire
[31]	Morrison, S.C. et al., 2013	Interventional	14 (14/0)	6–11 years	Children with DCD were at the top end of the scale for lower limb hypermobility.	Not specified	Lower Limb Assessment Score Foot Posture Index	6-Minute Walk Test GAITRite walkway
[32]	Celletti, C. et al., 2015	Observational	41	Mean age 8 ± 3 years	Children with DCD and GJH showed a significant excess of frequent falls, easy bruising, motor impersistence.	M-ABC VMI	Beighton score (≥5 for 3–9 years; ≥4 for >10 years)	Linguistic Comprehension Test, Peabody Picture Vocabulary Test, Boston Naming Test, Bus Story Test, and Memoria-Training tests WISC-IV
[33]	Engelbert et al., 2005	observational	56	4–12 years	No significant association between the presence of a delay in motor development and joint hypermobility	M-ABC	Bulbena criteria	-

M-ABC-2—Movement assessment battery for children-second edition; JHS—joint hypermobility syndrome; DCD—Developmental Coordination Disorder; ADHD—Attention-Deficit/Hyperactivity Disorder; HCTDs—hereditary connective tissue disorders; pDCD—probable developmental coordination disorder; GJH—generalized joint hypermobility; BJHS—benign joint hypermobility syndrome; PAL—Physical Activity Level; HMS—Hypermobility Syndrome.

## Data Availability

Data sharing not applicable.
